# Synchronously Predicting Tea Polyphenol and Epigallocatechin Gallate in Tea Leaves Using Fourier Transform–Near-Infrared Spectroscopy and Machine Learning

**DOI:** 10.3390/molecules28145379

**Published:** 2023-07-13

**Authors:** Sitan Ye, Haiyong Weng, Lirong Xiang, Liangquan Jia, Jinchai Xu

**Affiliations:** 1 School of Engineering, Newcastle University, Newcastle upon Tyne NE1 7RU, UK; yesitan@outlook.com; 2 Fujian Key Laboratory of Agricultural Information Sensoring Technology, College of Mechanical and Electrical Engineering, Fujian Agriculture and Forestry University, Fuzhou 350002, China; hyweng@fafu.edu.cn; 3 School of Future Technology, Haixia Institute of Science and Technology, Fujian Agriculture and Forestry University, Fuzhou 350002, China; 4 Department of Biological and Agricultural Engineering, North Carolina State University, Raleigh, NC 27606, USA; 5 School of Information Engineering, Huzhou University, Huzhou 313000, China

**Keywords:** tea polyphenol, EGCG, Fourier Transform–near-infrared spectroscopy, machine learning, rapid prediction

## Abstract

Tea polyphenol and epigallocatechin gallate (EGCG) were considered as key components of tea. The rapid prediction of these two components can be beneficial for tea quality control and product development for tea producers, breeders and consumers. This study aimed to develop reliable models for tea polyphenols and EGCG content prediction during the breeding process using Fourier Transform–near infrared (FT-NIR) spectroscopy combined with machine learning algorithms. Various spectral preprocessing methods including Savitzky–Golay smoothing (SG), standard normal variate (SNV), vector normalization (VN), multiplicative scatter correction (MSC) and first derivative (FD) were applied to improve the quality of the collected spectra. Partial least squares regression (PLSR) and least squares support vector regression (LS-SVR) were introduced to establish models for tea polyphenol and EGCG content prediction based on different preprocessed spectral data. Variable selection algorithms, including competitive adaptive reweighted sampling (CARS) and random forest (RF), were further utilized to identify key spectral bands to improve the efficiency of the models. The results demonstrate that the optimal model for tea polyphenols calibration was the LS-SVR with R_p_ = 0.975 and RPD = 4.540 based on SG-smoothed full spectra. For EGCG detection, the best model was the LS-SVR with R_p_ = 0.936 and RPD = 2.841 using full original spectra as model inputs. The application of variable selection algorithms further improved the predictive performance of the models. The LS-SVR model for tea polyphenols prediction with R_p_ = 0.978 and RPD = 4.833 used 30 CARS-selected variables, while the LS-SVR model build on 27 RF-selected variables achieved the best predictive ability with R_p_ = 0.944 and RPD = 3.049, respectively, for EGCG prediction. The results demonstrate a potential of FT-NIR spectroscopy combined with machine learning for the rapid screening of genotypes with high tea polyphenol and EGCG content in tea leaves.

## 1. Introduction

Tea, as one of the top three non-alcoholic beverages in the world, has received significant attention due to its numerous health benefits attributed to its rich content of bioactive compounds, particularly tea polyphenols and epigallocatechin gallate (EGCG) [1]. These bioactive compounds have been associated with various health-promoting effects, such as antioxidant, anti-inflammatory, antimicrobial, and anticancer properties [2]. The accurate and rapid detection of tea polyphenols and EGCG content in various tea varieties is crucial for quality control, product development, and consumer preferences. Traditional methods such as the Folin phenol method and high-performance liquid chromatography (HPLC) are time-consuming, labor-intensive, and require sample destruction [3]. Therefore, there is a need for alternative methods that allow the rapid and reliable detection of tea polyphenols and EGCG content in different tea varieties during the screening process.

Spectroscopy is a non-destructive analytical technique that has shown great potential in various fields, including agriculture, food science, and pharmaceuticals, for the rapid and accurate detection of chemical constituents in complex matrices [4,5,6]. The use of Spectroscopy in combination with chemometrics has been demonstrated to offer reliable and accurate predictions of various chemical components in complex samples [7,8]. In previous research, spectral technology was widely used in the monitoring of tea quality. The use of visible near-infrared spectroscopy technology was employed to detect the content of caffeine during the processing of green tea. The sensitive wavebands were extracted by SPA, and qualitative and quantitative models were established. The results show that the SPA-MLR mode had better predictive performance in detecting the content of tea polyphenols and caffeine, with a determination coefficient of prediction (Rp^2^) greater than 0.834 [9]. Kumar et al. (2018) used near-infrared spectroscopy technology, and a rapid detection of the content in fresh tea leaves was established through PLSR. Regression analysis was performed on the near-infrared spectroscopy data and tea polyphenols contents of 55 samples. The results show that the established PLSR model can accurately predict the tea polyphenols content of fresh tea leaves, with an Rp^2^ greater than 0.95 [3]. Lee et al. (2014) used NIR spectroscopy to collect spectral data from green tea powder and combined this with HPLC to determine the contents of green tea caffeine and nine catechin monomers (EGCG, EGC and GC, etc.); they constructed quantitative models based on modified partial least-squares (MPLS), principal component regression (PCR), and multiple linear regression (MLR) using the near-infrared spectroscopy data and the internal substances in green tea powder. The results show that the Rp^2^ of the MPLS model for the major catechin monomers (EGCG, EGC, etc.) and caffeine were all greater than 0.90, while those for gallocatechin (GC) were less than 0.81 [10]. Chen et al. (2021) employed a visible and near-infrared (Vis/NIR) spectrometer to accumulate spectral data from tea leaves throughout the fermentation process. The modified MPLS model they developed exhibited a modeling determination coefficient of calibration (Rc^2^) exceeding 0.94 for both total catechins and theanine contents [11]. The above results demonstrate the feasibility of applying spectroscopic techniques in tea quality testing; however, this approach is rarely used for the rapid detection of tea polyphenols and EGCG content during breeding process.

Under these scenarios, the expected outcomes of this research include a comprehensive understanding of the distribution of tea polyphenols and EGCG content in various tea varieties during the breeding progress, and developing models for rapidly predicting tea polyphenols and EGCG content within tea leaves. The findings of this study will provide a guideline for the rapid detection of these bioactive compounds in tea leaves and contribute to the existing knowledge on tea polyphenols and EGCG content in different tea varieties during the breeding process.

## 2. Results and Discussion

### 2.1. Statistical Analysis of Tea Polyphenols and EGCG Content in Different Varieties

**Figure 1:**
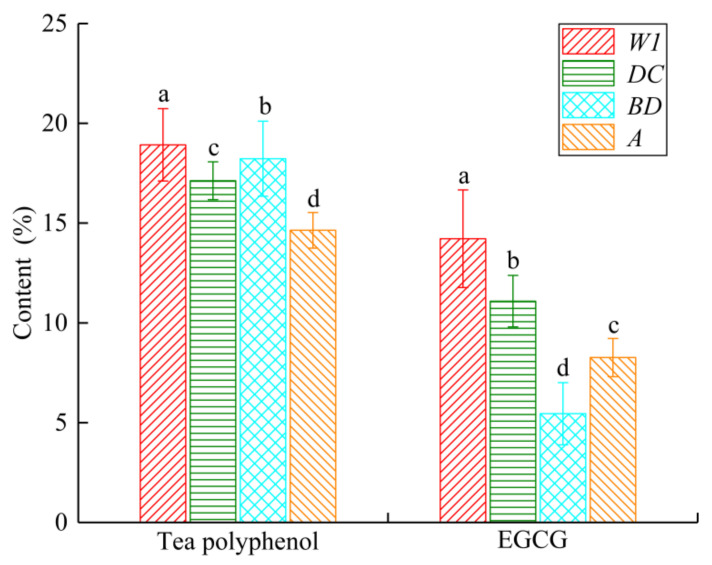
Analysis of tea polyphenol and EGCG contents of different varieties of tea leaves. Data are shown as mean ± standard deviation (*n* = 30). Different letters indicate significant difference at *p* < 0.05 based on Duncan test. *W1*, *DC*, *BD* and *A* stand for four the different types of tea tree variety, respectively.

The contents of tea polyphenol and EGCG contents of 84 samples are shown in Figure 1. The mean contents of tea polyphenol and EGCG in the four varieties were 15.54 ± 2.29% and 8.73 ± 2.75%, respectively. It can be seen that the EGCG content gradient is larger than of tea polyphenol. The *p*-values of the tea polyphenol and EGCG contents are 3.779 × 10^−11^ and 3.375 × 10^−14^, correspondingly, and the observed values were less than 0.05, thereby illustrating that the variations in tea polyphenol and EGCG content among different tea varieties are statistically significant. These findings serve as foundational support for the development of a robust and reliable detection model.

### 2.2. Analysis of Fourier Transform–Near-Infrared Spectroscopy Curves of Tea Powder

**Figure 2:**
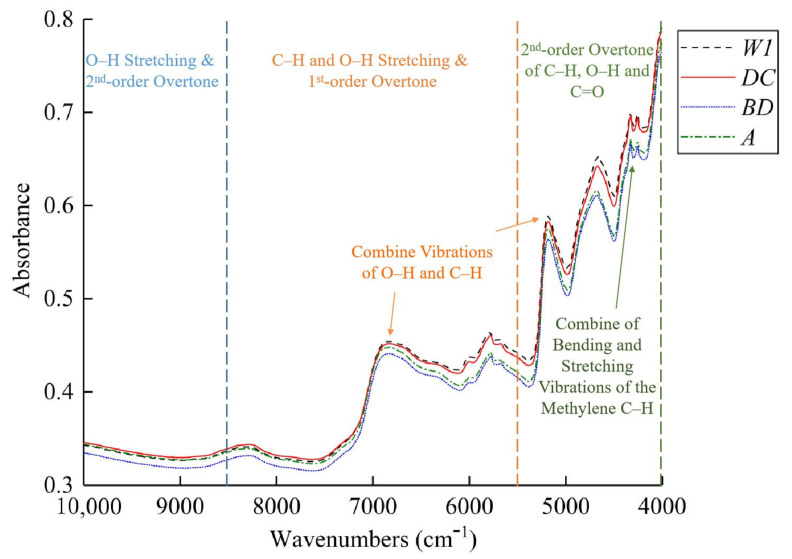
Average spectral reflectance of different varieties of tea powder.

FT-NIR spectra of tea powder samples are shown in Figure 2. As corroborated by prior scholarly investigations, the near-infrared spectral region communicates both the overtone and combination absorption data associated with the stretching vibrations of the hydrogen-based groups present in the organic constituents of the samples under study. In the range of 10,000–8500 cm^−1^, the spectral information is mainly related to the second-order overtone and a combination of the stretching vibrations of the O–H group [12]. In the range of 8500–5500 cm^−1^, the prominent absorption peaks are mainly due to the first-order overtone and a combination of the stretching vibrations of C–H and O–H groups [13]. Within the spectral range of 5500–4000 cm^−1^, an increased prevalence of absorption bands is observed, attributable to the second-order overtones of C–H and O–H groups, along with C=O bonds [14]. Thus, these absorption bands are closely related to the O–H and C–H groups in phenolic substances. In near-infrared spectroscopy, there were two obvious absorption bands at 5170 cm^−1^ and 6690 cm^−1^, mainly due to the combination vibrations of O–H and C–H groups. The absorption band at 4430 cm^−1^ is caused by the combination of bending and stretching vibrations of the methylene C–H group [15]. It can be seen that within the range of 10,000–4000 cm^−1^, the trends of the near-infrared spectral curves of different samples were similar, as well as the positions of the absorption peaks. However, the absorbance magnitudes varied, indicating different contents of tea polyphenols and EGCG in different samples.

### 2.3. Outliers Elimination

**Figure 3:**
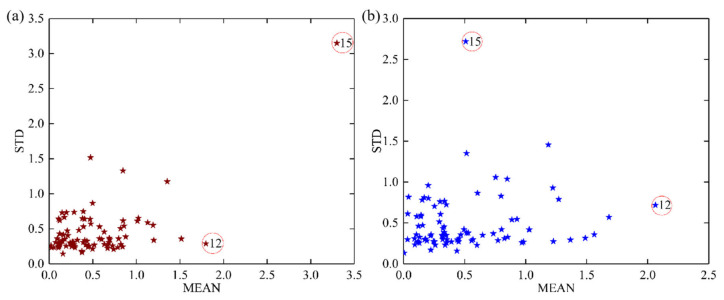
MEAN-STD distribution of tea polyphenol content (**a**) and EGCG content (**b**). The stars with different colors represent the predicted residuals of different key components of tea, where red stars represents the content of tea polyphenol and blue stars represents the EGCG content. Stars with red circles indicate the eliminated sample.

Outlier elimination was carried out before establishing a robust model for the rapid prediction of tea polyphenols and EGCG. In this study, the PLSR model was constructed with a number of iterations of 1000. After the iteration, the prediction residuals of all samples were obtained, and the MEAN-STD distribution was plotted as shown in Figure 3. Upon evaluation, it is discernible that certain samples exhibit elevated mean values and high standard deviations, deviating significantly from the core sample group. These anomalies are identified as outliers requiring elimination prior to the construction of the predictive model, thereby ensuring the robustness and accuracy of the model is not compromised by these extreme values. The Monte Carlo cross-validation (MCCV) method was used to eliminate potential outliers based on a threshold, and to establish a PLSR model for testing. The threshold was set at four times the mean value of all samples. For tea polyphenol, samples had a mean (MEAN) and a standard deviation (STD) greater than 2.058 and 1.816, respectively. The results show that *sample 15* was regarded as a potential outlier. For EGCG, samples had a MEAN and STD greater than 2.062 and 1.967, respectively. We identified *sample 12* and *15* as potential outliers. This was because *sample 12* had abnormal EGCG content measurements but normal near-infrared spectral data, while *sample 15* presented the opposite pattern.

The prediction performance of the PLSR model before and after outlier elimination is shown in Table 1. It was found that the removal of potential outliers can improve the prediction performance of PLSR for both tea polyphenol and EGCG content. RPD = 3.721 for tea polyphenol and RPD = 1.981 for EGCG content, which increased by 11.24% and 10.30%, respectively. Therefore, *sample 12* and *15* were eliminated in advance for subsequent detection model construction to ensure the stability of model prediction.

### 2.4. Description of Sample for Model Establishment

Before constructing the prediction model, it is necessary to divide the sample set reasonably. The Kennard–Stone algorithm was used to divide the remaining 82 samples after outlier elimination into modeling and prediction sets at a ratio of 3:1. A total of 55 samples were obtained for the modeling set, with the remaining 27 samples as the prediction set. The modeling and prediction set data for tea polyphenol and EGCG content were statistically analyzed, and the specific results are shown in Table 2. It can be seen that the ranges of tea polyphenol and EGCG content in the training dataset were greater than that in the prediction set, and the distributions of these two components were uniform in both datasets, with similar mean values and standard deviations. Therefore, the division of the two chemical contents is reasonable for model establishment.

### 2.5. Models Establishment for Tea Polyphenol and EGCG Content Prediction

#### 2.5.1. Model Establishment Based on Full Spectrum

The process of collecting tea powder spectral data using an FT-NIR spectrometer, besides scanning the spectral information of the tea samples, also included irrelevant information such as instrument noise and stray light. In order to reduce the interference caused by noise in constructing the model and improve the signal-to-noise ratio of the spectral data, five pretreatment methods including SG-Smooth, SNV, VN, MSC, and FD were applied to the full spectra for analysis. The quantitative detection models for predicting tea polyphenol and EGCG content based on different pretreatments are shown in Table 3. In general, correlation coefficients of the different models for tea polyphenol content prediction were all greater than 0.955. Under the situation of the original spectra preprocessed using SG smoothing, the predictive ability of the LS-SVR model was improved, but that of PLSR was reduced. For using SNV, MSC, and VN, the predictive performance of PLSR and LS-SVR showed a downward trend. When FD preprocessing was applied, the predictive ability of the LS-SVR declined significantly, while that of PLSR model was improved. This indicates that different preprocessing methods have different adaptabilities to different detection models. Therefore, it was necessary to consider pretreatments and models simultaneously with the aim of building the most feasible model for tea polyphenol prediction. As seen from Table 3, the LS-SVR based on SG smoothing achieved the best results in terms of predicting ability, with R_p_ and RPD values of 0.975 and 4.540, respectively. Similarly, it can be seen that different preprocessing methods have different impacts on the EGCG prediction of different models. The LS-SVR model without preprocessing reached the best predictive performance, with R_p_ and RPD values of 0.936 and 2.841, respectively. In comparison to the model used for tea polyphenol prediction, the overall performance of the EGCG content prediction model was relatively low, which may be because the EGCG content was lower than that of tea polyphenol in the sample as it accounted for about 60% of total tea polyphenol prediction [16]. For EGCG content, the predictive abilities of PLSR and LS-SVR decreased after applying SG smoothing, but the predictive ability of the PLSR model improved when using the SNV, VN, MSC, and FD methods. Although the predictive performance of the LS-SVR model established after applying five preprocessing methods was lower than that of the LS-SVR model using the original spectrum, the R_p_ values of these models were all greater than 0.916. Therefore, further analysis for EGCG content prediction focused on a combination of the original spectrum and LS-SVR model.

#### 2.5.2. Model Establishment Based on Selected Sensitive Wavenumbers

**Figure 4:**
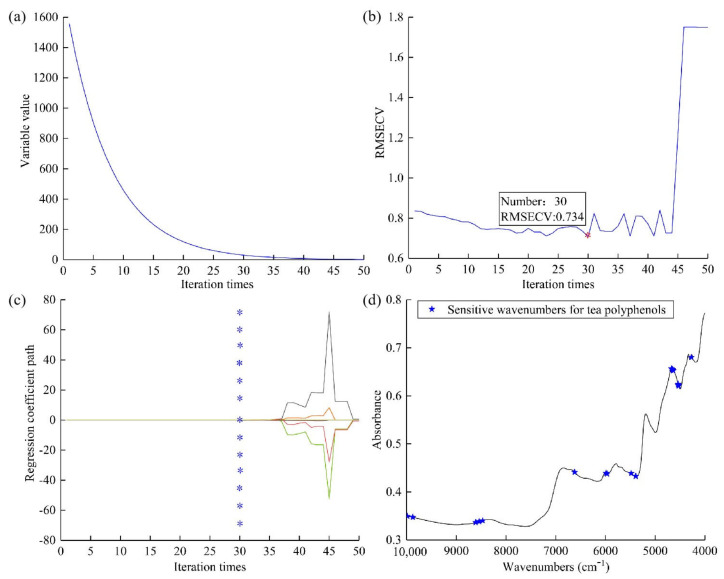
Selection results of CARS algorithm for tea polyphenol content. Trend of wavenumbers (**a**), RMSECV (**b**), and regression coefficient path (**c**), with the change of iteration times. Selected sensitive wavenumbers’ distribution situation (**d**). The regression coefficient for each variable, which one variable for each different color line, varies with the iteration times. “*” corresponds to the 30th sampling and RMSECV attained its minimum value.

**Figure 5:**
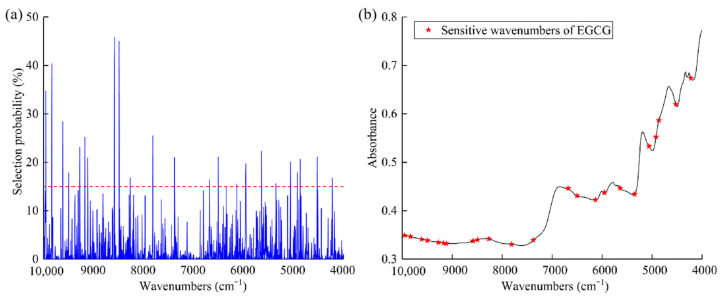
The Random Forest (RF) algorithm was utilized to extract the wavenumbers pertinent to EGCG content. The selection probability associated with each wavenumber is presented in (**a**), while the distribution of the initial 27 selected wavenumbers is depicted in (**b**). The red dotted line indicates a selection probability threshold was chosen to be 15%.

The models established based on full spectra achieved good prediction performances. However, a total of 1557 wavenumbers within the full spectra might contain some redundant spectral information. To simplify the detection model, sensitive wavenumber selection for tea polyphenols and EGCG prediction was carried out using CARS and RF. The distribution of these sensitive wavenumbers for tea polyphenol prediction in the FT-NIR spectrum is shown in Figure 4. The Monte Carlo (MC) sampling frequency was set at 1000 for the CARS algorithm, using the root mean square error method for five-fold cross-validation. With the increase in sampling frequency, the number of selected variables declined following an exponential decay function. At a sampling frequency of 50, only two variables remained, as illustrated in Figure 5a. When the 30th sampling instance was reached, the root mean square error of cross-validation (RMSECV) attained its minimum value of 0.734. This result suggests that variables unrelated to tea polyphenol content and variables that were collinear have been effectively eliminated.

For tea polyphenol, the 30 sensitive wavenumbers (4273, 4528, 4531, 4535, 4539, 4636, 4639, 4643, 4647, 4651, 4670, 4674, 5388, 5391, 5395, 5484, 5966, 5970, 5989, 6618, 6622, 8469, 8539, 8543, 8608, 8612, 9873, 9877, 9985, and 9989 cm^−1^) selected by the CARS algorithm were used for the LS-SVR establishment of tea polyphenol content prediction. In the FT-NIR spectral region, these 30 sensitive wavenumbers of tea polyphenols prediction were attributed to the O–H and C–H groups, and C-C absorptions in the phenolic ring [17]. The sensitive wavenumbers extracted by the CARS algorithm were mainly divided into three spectral regions: 4273–5484 cm^−1^, 5966–6622 cm^−1^ and 8469–9989 cm^−1^. Within 4273–4674 cm^−1^, the 12 sensitive wavenumbers (4273, 4528, 4531, 4535, 4539, 4636, 4639, 4643, 4647, 4651, 4670, and 4674 cm^−1^) were attributed to the methylene C–H, phenolic O–H, and phenolic C=O groups [18]. Within 5966–6622 cm^−1^, the nine sensitive wavelengths (5388, 5391, 5395, 5484, 5966, 5970, 5989, 6618, and 6622 cm^−1^) were attributed to the aromatic C–H and O–H groups [19]. Within 8469–9989 cm^−1^, the nine sensitive wavenumbers (8469, 8539, 8543, 8608, 8612, 9873, 9877, 9985, and 9989 cm^−1^) are attributed to the C–H stretching vibrations and phenolic O–H groups [20]. The wavenumber near 4273 cm^−1^ was related to the combination of the methylene C–H overtone stretching vibration and bending vibration [21]. The wavenumber near 4651 cm^−1^ was due to the combination of stretching vibrations of tertiary and primary amines [22]. The wavenumber near 8469 cm^−1^ was caused by the second overtone of the methylene C–H stretching vibration [6].

For EGCG, The LS-SVR model established using sensitive wavenumbers extracted by the RF algorithm based on the original spectrum is suitable for EGCG content prediction. The number of iterations of the RF algorithm was set to 1000, and a probability threshold of 15% was chosen to select the first 27 sensitive wavenumbers with a higher probability, based on the fact that a higher probability indicates that the wave number is more critical. The 27 sensitive wavenumbers (4223, 4524, 4863, 4921, 5060, 5349, 5638, 5951, 5955, 5958, 6132, 6502, 6680, 7378, 7814, 8265, 8489, 8581, 8585, 9117, 9175, 9275, 9499, 9615, 9835, 9839, and 9954 cm^−1^) are shown in Figure 5. In the FT-NIR spectral region, the 27 sensitive wavenumbers of the EGCG functional groups were attributed to the O–H and C–H groups in phenolics, and C=O in lipids [23]. The four sensitive wavenumbers extracted by the RF algorithm within 4223–4921 cm^−1^ (4223, 4524, 4863 and 4921 cm^−1^) were attributed to the first combination of frequencies caused by -CH_2_ groups [24]. The three sensitive wavenumbers within 5060–5638 cm^−1^ (5060, 5349 and 5638 cm^−1^) were attributed to C–H related to free -OH groups and methylene [22]. The five sensitive wavenumbers within 5951–6680 cm^−1^ (5951, 5955, 5958, 6132, 6502, 6680, 7378 and 7814 cm^−1^) were attributed to the C–H group of aromatic hydrocarbons [25]. The four sensitive wavenumbers within 8265–8585 cm^−1^ (8265, 8489, 8581 and 8585 cm^−1^) were attributed to second-order multiples of the C–H stretching vibration in -CH_2_ [20]. The eight sensitive wavenumbers within 9117–9954 cm^−1^ (9117, 9175, 9275, 9499, 9615, 9835, 9839 and 9954 cm^−1^) were attributed to the second-order multiples of the bound O–H group [26].

For detecting tea polyphenol content, the combination of the CARS algorithm and the LS-SVR model after SG smoothing preprocessing yielded an R_p_ value over 0.97 and an RPD of 4.833. It used only 30 wavelengths, which can reduce the variables by 98.07% compared with the full 1557 wavelengths (Table 4). This indicates that the CARS algorithm can effectively extract the key bands for detecting tea polyphenol content and eliminate irrelevant or multicollinear variables. For EGCG content detection, the combination of the RF algorithm and the LS-SVR model had the best performance, with R_p_ and RPD values of 0.944 and 3.049, respectively, using 27 wavelengths, which can reduce the variables by 98.26%. This suggests that the RF algorithm can also effectively extract the sensitive wavenumbers for EGCG prediction. The improved predictive performance of the models may be due to the reduction of irrelevant variables, resulting in a smaller number of independent variables. The findings suggest that the implementation of sensitive wavenumber selection effectively reduces the dimensionality of the input data and enhances the predictive capability of the model. This strategy could also be beneficial for swiftly identifying tea tree varieties with high tea polyphenol or EGCG content during the breeding process, thus accelerating the selection of superior cultivars.

## 3. Materials and Methods

### 3.1. Tea Powder Samples Preparation

**Figure 6:**
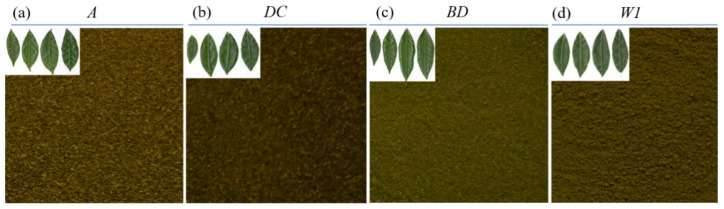
Fresh tea leaves and power of different tea varieties. *A* tea tree variety (**a**), *DC* tea tree variety (**b**), *BD* tea tree variety (**c**), and *W1* tea tree variety (**d**).

In this experiment, four species of tea trees were selected, which were *A*, *DC*, *BD*, and *W1* (*Camellia sinensis* L.), planted in the experimental garden of the Fujian Agriculture and Forestry University for screening genotypes with high tea polyphenols and EGCG contents within leaves. Fresh tea leaves were harvested from 28 to 31 March 2021. A total of 2520 fresh tea leaves were finally collected. The fresh tea leaves of different species are shown in Figure 6. Subsequently, 30 fresh tea leaves from each species were considered as one sample. The samples were then placed in an oven at 120 °C for 6 min for fixation, and then 90 °C for drying until constant weight. Finally, the dried samples were ground for 3 min in a multi-sample tissue grinder and pushed through an 80-mesh sieve to obtain tea powder as shown Figure 6. A total of 84 tea powder samples was finally obtained for this study.

### 3.2. Fourier Transform Near-Infrared Spectroscopy Data Collection

In this study, a Fourier Transform near-infrared spectrometer (Antaris II, Thermo Fisher Scientific, US) was used for spectral information collection. Before collecting spectral data, the spectrometer was preheated for half an hour to ensure a stable scanning state. Then, using the integrating sphere diffuse reflectance sampling module, approximately 3 g of tea powder was loaded into a sample cup rotator with an inner diameter of 4.78 cm. The sample cup was shaken to cover the bottom detection surface with tea powder before the sample was ready for testing. The instrument parameters were set to 64 scans, with a gain of 2 in the room temperature of approximately 25 °C. The background spectrum was removed and air was used as a reference. For each tea powder sample, near-infrared spectra were scanned at three different positions 120° apart at the bottom of the powder. The average of these three sets of spectral data was taken for analysis. After collecting the sample spectra, the tea powder was used for chemical content detection. Meanwhile, the sample cup was cleaned and prepared for the next collection.

### 3.3. Determination of Tea Polyphenol and EGCG Content

The tea powder samples’ Fourier Transform–near-infrared spectroscopy data were subjected to the determination of tea polyphenol and EGCG content. In this study, the Folin phenol method was used to determine the tea polyphenol content in the tea powder samples [27], and ultra-high-performance liquid chromatography (UPLC) was used to determine the EGCG content in accordance with the Chinese national standard GB/T 8313-2018 [4]. UPLC chromatographic detection conditions: the column used in the liquid chromatography was C18; flow rate of phase A and B—1 mL/min; column pressure—8650 psi; column temperature—35 °C; injection volume—2 μL; detector wavelength range—200–400 nm; detection wavelength—278 nm; scan duration—10 min. The gradient elution conditions for the liquid chromatographic mobile phase are shown in Table 5.

### 3.4. Data Analysis

#### 3.4.1. Preprocessing Methods

In this research, the following five different preprocessing methodologies were employed to prepare spectral data for subsequent analysis before predicting the content of EGCG and tea polyphenols in tea leaves. Savitzky–Golay smoothing (SG) performed a least squares fit of a small window of data to a polynomial of a certain degree, which preserved the features of the underlying signal while reducing noise [5]. The standard normal variate (SNV) transformation method is primarily employed to standardize individual spectra. This process ensures that each spectrum possesses a zero mean and a standard deviation unit of one. This normalization is accomplished by computing the mean of each spectrum, subtracting this mean value from the spectral data, and subsequently dividing by the standard deviation of the same spectrum [6]. The advantage of SNV is that it helps to correct for multiplicative scatter effects and other physical phenomena that can affect the light scatter properties of the observed spectrum [7]. Vector normalization (VN) was used to minimize the effect of illumination differences in the hyperspectral data, which can eliminate the influence of light intensity and only preserve the spectral shape information [8]. Multiplicative scatter correction (MSC) was used to correct for scale and offsets in the data, which was done by fitting a line to each individual spectrum and then adjusting the spectrum to match a standard or reference [28]. The line was fitted using linear regression; the slope of the line represents the scale, and the intercept represents the offset [29]. By adjusting each spectrum to match the standard, MSC can make the data more consistent and easier to analyze. The first derivative (FD) was to enhance small spectral features and differences between similar materials, making them more distinguishable [30]. Derivative spectroscopy involves the calculation of the rate of change of the reflectance or absorbance values with respect to the wavelength; it can help highlight the slopes of spectral features, which correspond to the absorption and emission characteristics of different materials [31].

#### 3.4.2. Prediction Models’ Establishment

In the context of detecting tea polyphenol and EGCG content, partial least squares regression (PLSR) and least squares support vector regression (LS-SVR) were used to establish models based on spectral data. PLSR is a multivariate regression method used for modeling relationships between sets of observed variables by means of latent variables [32]. It is particularly useful when the variables are highly collinear, when the number of observations is smaller than the number of variables, or when there is noise in the data [33]. LS-SVR is a multivariate regression method that can be used to analyze the relationships between two sets of observed variables [34]. LS-SVR is a variant of support vector machines (SVM), a set of machine learning methods typically used for classification, regression, and outlier detection [35].

#### 3.4.3. Models Performance Evaluation

In this study, the parameters of correlation coefficient (*R*), root mean square error (*RMSE*) and residual predictive deviation (*RPD*) were used for evaluating model performance. The larger values of the *R* and *RPD* and the smaller value of the *RMSE* indicated a better modeling performance [36]. These model performance indexes were defined using Equations (1)–(3) as follows:

(1) $$R=\frac{\sum_{i=1}^{n}\left(y_{i,a}-{\bar{y}}_{i,a}\right)\left(y_{i,p}-{\bar{y}}_{i,p}\right)}{\sqrt{\sum_{i=1}^{n}{\left(y_{i,a}-{\bar{y}}_{i,a}\right)}^{2}}\;\sqrt{\sum_{i=1}^{n}{\left(y_{i,p}-{\bar{y}}_{i,p}\right)}^{2}}}$$

(2) $$RMSE=\sqrt{\frac{\sum_{i=1}^{n}{\left(y_{i,p}-{\bar{y}}_{i,p}\right)}^{2}}{n-1}}$$

(3) $$RPD=\frac{SD_{v}}{SEP}=\frac{1}{\sqrt{1-R^{2}}}$$

where $y_{i,a},\;y_{i,p}$ are the actual measured chemical values and the predicted chemical values of sample *i*; ${\bar{y}}_{i,a},\;{\bar{y}}_{i,p}$ are the average actual measured chemical values and the average predicted chemical values of the sample; $SD_{v},\;SEP$ are the standard deviation of sample content in the prediction set and the standard deviation of the predictions; n is the number of samples.

#### 3.4.4. Software and Statistical Analyses

Spectral data processing in this study was carried out using Matlab 2016a (The Math Works, Natick, MA, USA). The Unscrambler X10.1 (CAMO AS, Oslo, Norway) was used for data preprocessing. Origin 2017C (OriginLab, Northampton, MA, USA) was used for data illustration in graphs.

## 4. Conclusions

In this study, models for rapidly predicting tea polyphenols and EGCG within tea leaves during the breeding process based on Fourier Transform–near-infrared spectroscopy (10,000–4000 cm^−1^) were developed. The distributions of tea polyphenols and EGCG content in four tea tree varieties and their spectral response characteristics were analyzed. Detection models for tea polyphenols and EGCG content were established based on full-band spectral preprocessing. To simplify the model and improve its computation speed, two variable selection algorithms were combined with machine learning to predict the tea polyphenols and EGCG content. The results show that the LS-SVR model established based on 30 sensitive spectral bands selected by the CARS algorithm obtained a good result for tea polyphenols prediction, with an R_p_ value of 0.978 and an RPD of 4.833. The LS-SVR model trained on 27 sensitive spectral bands selected by the RF algorithm for EGCG prediction achieved an R_p_ value of 0.944 and an RPD of 3.049, respectively. The results demonstrate that Fourier Transform–near-infrared spectroscopy combined with machine learning enables the rapid prediction of tea polyphenols and EGCG content in tea leaves.

## Figures and Tables

**Table 1:** Performance of PLSR before and after removing outliers.

Content	*n*	R_c_	RMSEC (%)	R_p_	RMSEP (%)	RPD
Tea Polyphenol	0	0.951	0.516	0.954	0.568	3.345
	2	0.979	0.464	0.963	0.545	3.721
EGCG	0	0.982	0.580	0.830	1.003	1.796
	2	0.980	0.591	0.863	0.904	1.981

Note: The variable *n* means the number of samples being removed. The notations R_c_ and R_p,_ respectively, represent the correlation coefficients corresponding to the calibration set and the prediction set. Similarly, the acronyms RMSEC and RMSEP are utilized to denote the root mean square error within the calibration set and prediction set, respectively. RPD is an abbreviation used to refer to the residual predictive deviation, a metric used in model evaluation.

**Table 2:** Distribution of tea polyphenols and EGCG content in sample sets.

	Tea Polyphenols Content		EGCG Content	
	Calibration Set	Prediction Set	Calibration Set	Prediction Set
*N*	55	27	55	27
Range (%)	11.17–21.96	12.62–18.82	3.38–18.43	5.72–11.68
Mean (%)	15.88	14.64	8.90	8.18
STD (%)	2.33	1.88	3.07	1.51

Note: *N* represents the number of samples. STD represents standard deviation.

**Table 3:** The performance of quantitative models for tea polyphenol and EGCG content prediction based on full waveband under different pretreatments.

	Model	Preprocessing	R_c_	RMSEC (%)	R_p_	RMSEP (%)	RPD
	PLSR	None	0.979	0.464	0.963	0.545	3.721
		SG-Smooth	0.979	0.466	0.963	0.546	3.715
		SNV	0.981	0.443	0.963	0.543	3.711
		VN	0.979	0.468	0.959	0.546	3.563
		MSC	0.981	0.445	0.961	0.549	3.644
Tea		FD	0.992	0.280	0.963	0.571	3.724
Polyphnenol	LS-SVR	None	0.980	0.449	0.975	0.420	4.539
		SG-Smooth	0.980	0.449	0.975	0.420	4.540
		SNV	0.977	0.490	0.964	0.503	3.802
		VN	0.980	0.449	0.972	0.438	4.322
		MSC	0.977	0.490	0.964	0.505	3.792
		FD	0.999	0.443	0.955	0.578	3.389
	PLSR	None	0.980	0.591	0.863	0.904	1.981
		SG-Smooth	0.980	0.592	0.863	0.904	1.980
		SNV	0.982	0.569	0.913	0.661	2.461
		VN	0.985	0.509	0.898	0.731	2.280
		MSC	0.983	0.555	0.909	0.678	2.402
EGCG		FD	0.992	0.369	0.918	0.661	2.521
	LS-SVR	None	0.993	0.361	0.936	0.637	2.841
		SG-Smooth	0.992	0.363	0.935	0.638	2.839
		SNV	0.993	0.337	0.922	0.682	2.587
		VN	0.988	0.454	0.934	0.542	2.807
		MSC	0.994	0.320	0.916	0.709	2.506
		FD	0.998	0.236	0.925	0.681	2.646

Note: γ = 12,693.4 and δ^2^ = 23,784.6 for SG-LS-SVR for tea polyphenol prediction, and γ = 113,732.9 and δ^2^ = 31,844.048 for SG-LS-SVR for EGCG prediction.

**Table 4:** Tea polyphenol and EGCG content prediction under different variable selection methods.

Content	Model	Number	R_c_	RMSEC (%)	R_p_	RMSEP (%)	RPD
Tea Polyphenol	SG-Smooth-CARS-LS-SVR	30	0.984	0.404	0.978	0.395	4.833
	SG-Smooth-RF-LS-SVR	16	0.975	0.504	0.926	0.716	2.655
EGCG	None-CARS-LS-SVR	20	0.995	0.306	0.901	0.796	2.315
	None-RF-LS-SVR	27	0.996	0.267	0.944	0.937	3.049

**Table 5:** Gradient elution conditions for liquid chromatography mobile phases.

Time (min)	Mobile Phase A (%)	Mobile Phase B (%)	Injection Volume (μL)
0.0	93	7	0.4
1.5	93	7	0.4
4.5	74	26	0.4
8.0	68	32	0.4
10.0	93	7	0.4

## Data Availability

The data and programming codes are freely available upon request.
